# Empirical evaluation of humpback whale telomere length estimates; quality control and factors causing variability in the singleplex and multiplex qPCR methods

**DOI:** 10.1186/1471-2156-13-77

**Published:** 2012-09-06

**Authors:** Morten Tange Olsen, Martine Bérubé, Jooke Robbins, Per J Palsbøll

**Affiliations:** 1 Evolutionary Genetics Group, Department of Genetics, Microbiology, and Toxicology, Stockholm University, Stockholm, S-106 91, Sweden; 2 Marine Evolution and Conservation, Centre for Ecological and Evolutionary Studies, University of Groningen, PO Box 11103, 97 CC, Groningen, The Netherlands; 3 Provincetown Center for Coastal Studies, 5 Holway Avenue, Provincetown, MA, 02657, USA; 4 Section for Evolutionary Genomics, Centre for GeoGenetics, Natural History Museum of Denmark, University of Copenhagen Øster Voldgade 5-7, Copenhagen K 1350, Denmark

**Keywords:** Quantitative PCR, Telomere length, Quality control, Non-model species, Guidelines

## Abstract

**Background:**

Telomeres, the protective cap of chromosomes, have emerged as powerful markers of biological age and life history in model and non-model species. The qPCR method for telomere length estimation is one of the most common methods for telomere length estimation, but has received recent critique for being too error-prone and yielding unreliable results. This critique coincides with an increasing awareness of the potentials and limitations of the qPCR technique in general and the proposal of a general set of guidelines (MIQE) for standardization of experimental, analytical, and reporting steps of qPCR. In order to evaluate the utility of the qPCR method for telomere length estimation in non-model species, we carried out four different qPCR assays directed at humpback whale telomeres, and subsequently performed a rigorous quality control to evaluate the performance of each assay.

**Results:**

Performance differed substantially among assays and only one assay was found useful for telomere length estimation in humpback whales. The most notable factors causing these inter-assay differences were primer design and choice of using singleplex or multiplex assays. Inferred amplification efficiencies differed by up to 40% depending on assay and quantification method, however this variation only affected telomere length estimates in the worst performing assays.

**Conclusion:**

Our results suggest that seemingly well performing qPCR assays may contain biases that will only be detected by extensive quality control. Moreover, we show that the qPCR method for telomere length estimation can be highly precise and accurate, and thus suitable for telomere measurement in non-model species, if effort is devoted to optimization at all experimental and analytical steps. We conclude by highlighting a set of quality controls which may serve for further standardization of the qPCR method for telomere length estimation, and discuss some of the factors that may cause variation in qPCR experiments.

## Background

Telomeres play a key role in maintaining chromosome integrity and are crucial for normal cell function [1]. In vertebrates, telomeres consist of tandem repeated TTAGGG sequences at the end of chromosomes [2]. Telomere repeats are lost during cell replication and by oxidative damage [3-5], and telomeres thus tend to shorten with age, ultimately reaching a threshold which likely contributes to cellular and organismal senescence [6-10].

The predicted loss of telomere repeats with age, and observations that the rate at which this happens correlate with fitness, lifespan, and susceptibility to a range of diseases, have sparked a general interest in understanding the role of telomere dynamics in life histories [11-20], as well as using telomeres as a molecular tool for determination of chronological and biological age in non-model species [21-23].

One approach to telomere length estimation is the qPCR method developed by Cawthon [24,25]. Targeting telomere repeat sequences in a qPCR assay is possible by the use of specially designed primers with build-in mismatches that allow for binding and amplification of the telomere target, but not amplification of primer dimers [24,25]. In qPCR, a DNA-binding fluorescence-dye such as SYBR green is used to monitor the amplification in individual PCR reactions and determine the point in time during cycling, the C_q_ value, when amplification of target crosses a fixed threshold. The resulting sample C_q_ values can be translated into estimates of the amount of telomere repeats (T) in a sample by means of a standard curve under the assumption that the amount of fluorescence directly correlate with the amount of double-stranded DNA that is amplified. This T can be scaled against the amount of a single copy reference gene (S) to obtain the T/S ratio, which is an estimate of the relative amount of telomere repeats in the sample.

The general speed, sensitivity, and conceptual and practical simplicity of the PCR technique have resulted in qPCR becoming the touchstone for nucleic acid quantification and comparison in several disciplines [26,27], and likewise, the telomere qPCR approach to telomere length estimation have become one of the most common methods for studying telomere dynamics.

However, despite its conceptual transparency and practical simplicity, obtaining, analyzing, and interpreting qPCR data is not a trivial issue. In particular, the high sensitivity of the technique implies that results may be of low precision and/or misleading if the qPCR assay is not adequately optimized. These problems have been exacerbated by the wide applicability and popularity of the technique along with a lack of general guidelines for how to perform and report qPCR experiments. As a consequence “*qPCR has become an inadequately standardized, complex, and frequently, inconsistent technique that invites the publication of flawed conclusions*” [28]. A similar critique has been directed towards the qPCR approach to telomere measurement and there is a growing debate about its research applications [29-33].

Still, there are several reasons why telomere estimation by the qPCR method may be attractive. First, many of the above issues are not inherent characteristics of the qPCR technique but mainly result from unfamiliarity with its technical requirements and limitations. This issue has been addressed by the recent formulation of the MIQE guidelines (Minimum Information for publication of Quantitative real-time PCR Experiments) with the purpose to “*ensure the integrity of the scientific literature, promote consistency between laboratories, and increase experimental transparency*” [26-28]. Second, scientific progress is made by developing, testing and optimizing, rather than just criticizing, as also noted by Monaghan [29] and Smith and co-authors [33]. That is, estimation of measurement precision and accuracy may provide the information required to identify and reduce the factors causing variability, and hence optimize the qPCR method. Finally, until recently, the qPCR method provided the only avenue for measuring telomere length in skin biopsies from free-ranging and generally inaccessible species for which even little information on telomere dynamics, however preliminary, is of value.

Here, we evaluate the performance of four assays based on the qPCR methods described by Cawthon [24,25]. The four assays differed with respect to primers, reagents, qPCR platform, and experimental setup, and were all modified specifically for estimation of telomere lengths in humpback whale skin samples. Our goals were to;

i. Arrive at a reliable qPCR assay specifically tailored for measuring telomeres in humpback whale skin samples

ii.Build on the MIQE guidelines and work of Karlen and co-authors [34] to propose a set of quality controls which may serve for further standardization of the qPCR method

iii. Highlight a subset of the factors affecting the precision and accuracy of telomere length estimates by qPCR; and, if nothing else

iv. Prevent others from making the same mistakes as we did by reporting all relevant aspects of our workflow and results.

Our approach is unique in its use of skin samples from a large, free-ranging marine mammal and its comparison of several different qPCR assays. However, although the focus is that of telomere estimation in humpback whale skin samples, the factors causing variability and the principles of quality control are universal and should apply to many qPCR applications.

## Methods

### Sampling and DNA extraction

Skin samples were obtained from the Gulf of Maine humpback whale population by use of biopsy techniques [35,36] and subsequently stored at −20°C in 10% DMSO. Genomic DNA was extracted using a modified phenol-chloroform method [37] or the QIAGENTM DNeasy Blood and Tissue kit according to the manufacturer’s instructions and stored in TE buffer (10 mM Tris·Cl, 0.5 mM EDTA, pH 9.0) at −20°C. The DNA concentration of each extraction was measured using a Thermo Scientific NanoDrop 8000 and DNA quality assessed by gel-electrophoresis [37].

### Experimental design

Each qPCR batch (i.e. run or plate) consisted of three components; i) a serial dilution series prepared from a pool of DNA from six humpback whales, which was used to construct standard curves and formed the basis of the primer optimization and quality control (Table 1); ii) “unknown” humpback whale samples for which we measured telomere lengths and tested the performance of different quantitative methods; and iii) no template controls (NTC), which allowed for detection of potential contamination and/or primer dimer formation. Each step of the dilution series, each unknown sample, and the NTC were run in triplicate in each batch, and the same “unknowns” analyzed in each of the four different qPCR assays.

**Table 1:** Experimental setup of the four qPCR assays for relative telomere length estimation

**Reaction conditions**					**Primers**		**Standards**			**Telomere**		**Reference**	
**Assay**	**Platform**	**Type**	**Master mix**	**Duration**	**Tel**	**Ref**	**Batches**	**Dilutions**	**LDR**	**Cq**	**NTC**	**Cq**	**NTC**
I	ABI	Singleplex	Commercial	21	CW2006	BA 1998	7	5	4.5-72.0	17.6	34.9	33.5	40.0
II	RG	Multiplex	RC 2009	6	RC 2009	RC 2009	4	4	2.2-60.0	15.7	19.4	39.9	27.9
III	RG	Singleplex	RC 2009	5	RC 2009	RC 2009	4	4	2.2-60.0	14.1	15.5	37.4	36.2
IV	RG	Multiplex	Commercial	6	RC 2009	RC 2009	4	5	4.5-72.0	13.9	27.2	34.3	31.1

Duration = number of days it took to generate the data once assays had been optimized; BA = Bérubé and Aguilar [38]; CW 2006 = Callicot and Womack [52]; RC 2009 = Cawthon [24]; Batches = number of separate qPCR runs (plates); Dilutions = number of dilution steps; LDR = linear dynamic range (ng/reaction); Cq = the average Cq of the serially diluted standard with the lowest amount of DNA; NTC = the lowest Cq value observed for a NTC.

### Primer optimization

Primers were obtained from the literature [24,25,38] or designed using the AmplifX program [39]. Testing and optimization was performed in a stepwise approach in which we first performed conventional PCRs [40] to assess whether the amplicon was of the expected length, then Sanger sequenced [41] the amplicon to confirm primer specificity, and finally performed a series of qPCR reactions on each primer pair, in which primer concentrations, annealing temperatures, and template DNA concentrations were kept as variables. In each of these reactions, melting curves allowed for assessment of primer specificity [42]. The conventional PCR conditions varied across primer pairs, but generally consisted of initial heating at 94°C for 2 minutes, followed by 30–40 cycles of denaturation at 94°C for 30 seconds, annealing for 30 seconds, and extension at 72°C for 30–240 seconds, and completion at 72°C for 10 minutes. PCR products were separated by electrophoresis through 1.7% agarose gels (FMC, Inc.) in 1xTBE buffer [37]. Sequencing was performed using the ABI BigDyeTM Terminator v3.1 cycle sequencing kit and an ABI 3130 genetic analyzer according to the manufacturer’s protocol (Applied Biosystems Inc.). Quantitative PCR reactions were performed as detailed below.

### qPCR reactions

The first assay, Assay I, was a singleplex assay similar to that of Cawthon [43] except for using a commercial SYBR green master mix. PCR reaction volumes were 25 μl and consisted of 50% ABsoluteTM QPCR SYBR© Green Mix (Thermo Fisher Scientific, Inc.), 0.200 μM ROX dye, 2–12 ng template DNA, and 0.625 μM of each primer as listed in ( Additional file 1: Table S1). PCR amplifications were conducted using an ABI PRISMTM 7000 Sequence Detection System using the MicroAmpTM optical 96-well reaction plates with optical 8-cap strips (Applied Biosystems, Inc.). The thermal cycling profile was 95°C for 15 min followed by 40 cycles of 95°C for 15 s and 56°C annealing, extension for 1 min, and concluded with a dissociation profile for construction of melting curves. The PCR conditions for the α-lactalbumin gene were identical to those of the telomere amplification, except that the annealing and extension temperature was set at 58°C.

In assay II, we tested the multiplex approach presented by Cawthon [24]. Here, telomere primers have been designed to amplify a fixed-length PCR product with well-defined melting profile useful for subsequent evaluation, and the reference gene primers contain a CG-clamp which increases their melting temperature, allowing for separate amplification of telomere and reference gene. Reactions had a volume of 25 ul and contained 0.75 SYBR Green I (Invitrogen), 10 mM Tris–HCl (pH8.3), 50 mM KCl, 3 mM MgCl2, 0.2 mM of each dNTP, 1 mM DTT, 0.625U of AmpliTaqGold, 1 M betaine (U.S. Biochemicals), and 20 ng DNA. Telomere primers and the albumin reference gene primers were as described by Cawthon [24] with the exception that we used 500 nM of each telomere primer and 1300 nM of each albumin reference gene primer (Additional file 1: Table S1). Amplifications were performed in a QIAGEN Rotor-Gene Q (former Corbett Rotor-Gene 6000) qPCR cycler using the manufacturer’s 0.1 ml strip tubes and caps. Conditions were 95°C for 15 min, 2 cycles of 94°C for 15 s and 49°C for 15 s, 40 cycles of 94°C for 15 s, 62°C for 10 s, 74°C for 15 s with signal acquisition, 84°C for 10 s, 88°C for 15 s with signal acquisition, and concluded with a melting curve ramping from 72°C to 95°C, rising by 0.5°C in steps of 30 s.

Assay III was a repetition of assay II, but with telomere and reference amplified in separate singleplex reactions to assess potential reaction inhibition caused by multiplexing. That is, the telomere portion of the assay only received telomere primers, and the reference gene portion only received albumin primers. The telomere was amplified using 95°C for 15 min, 2 cycles of 94°C for 15 s and 49°C for 15 s, 40 cycles of 94°C for 15 s, 62°C for 10 s, 74°C for 15 s with signal acquisition, and the reference gene reaction conditions were 95°C for 15 min, 2 cycles of 94°C for 15 s and 49°C for 15 s, 40 cycles of 94°C for 15 s, 84°C for 10 s, and 88°C for 15 s with signal acquisition.

Finally, assay IV was similar to assay II, but using a commercial SYBR green master mix rather than Cawthons’ master mix protocol. Each qPCR reaction was conducted in a total volume of 25 μl consisting of 50% ABsoluteTM QPCR SYBR© Green Mix Plus ROX vial (Thermo Fisher Scientific, Inc.), 800 nM of each primer, and 20 ng of template DNA. Consumables, PCR platform, and settings were as in assay II.

### Processing of raw fluorescence data

Initial visual examination of the amplification curves were done in the ABI Prism 7000 SDS Software version 1.2.3 for assay I and the Rotor-Gene 6000 Series Software version 1.7.87 for assays II-IV. Baseline correction was performed in LinRegPCR version 12.16 [44-46] unless otherwise noted. We used the automatic “strict” baseline correction option to adjust for background fluorescence noise, but made manual adjustments based on the minimum level of fluorescence in the few instances where the program could not set the baseline automatically. The fluorescence threshold line for determination of Cq values was fixed among batches and set to cross the linear phase of the amplification curve during which amplification is exponential [47]. The linear phase, or window of linearity (W-o-L) in the LinRegPCR terminology, was initially set for all reactions in common, but individually adjusted if required to obtain a better fit (i.e. a higher R^2^) of the regression line to the linear phase of the amplification curve.

### Serial dilution series

#### Efficiency and quantitative methods

The observed amount of telomere and reference gene in each dilution step of the serial dilution series was determined by the methods described by Pfaffl [48] and Ruijter and co-authors [46] (Equations 17 in the Appendix). The two methods differ in the way the amplification efficiency E is estimated. In the Pfaffl method, efficiency is estimated from the slope of the standard curve (i.e. calibration curve), which describes the linear regression between the log amount DNA of the serial dilution series and the Cq values observed at each dilution (Additional file 2: Figure S1A, B). In the Ruijter method, amplification efficiency is estimated from the slope of the regression line fitted to the log linear phase of each amplification curve as implemented in the LinRegPCR software. The telomere and reference gene amplification efficiencies were obtained for each batch as an average of those individual amplification efficiencies that did not deviate by more than 5% from the average, as described in Ruijter and co-authors [46]. In addition, we estimated the average efficiency of the triplicate reactions to assess whether efficiency varied across the range of the dilution series.

Similar to the Pfaffl method, a “standard curve” can be produced for the Ruijter method as the linear regression between the log amount DNA of the serial dilution series and the starting concentration N0 observed at each dilution step. The slope and intercept of these standard curves allow us to estimate the observed amount of telomere and reference gene [47] (Equation 6 in the Appendix).

#### Precision, robustness, accuracy, and resolution

**Figure 1:**
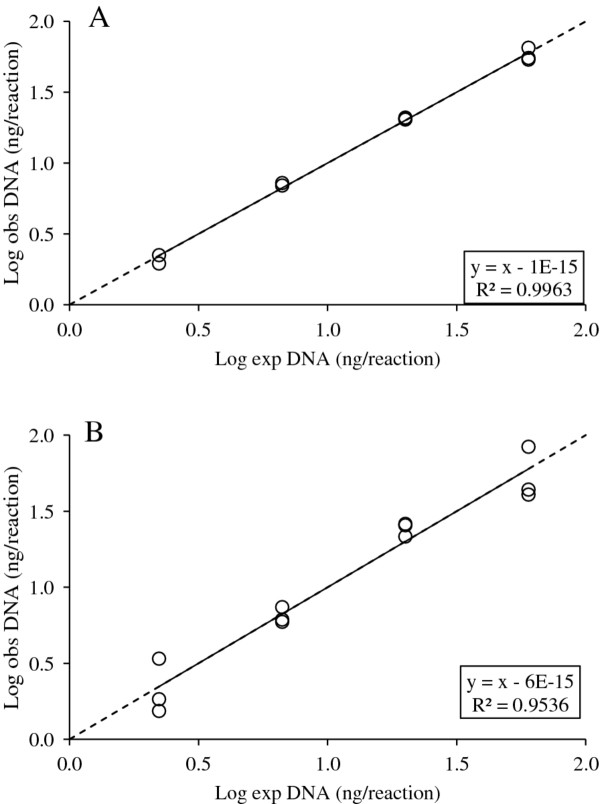
**Precision and accuracy illustrated by two reference gene batches from assay III where the expected input amount of DNA is plotted against the observed amount of DNA. **The solid line is the best fit through the observed amount DNA (circles) and the stippled line the expected fit when y = x.

Precision refers to the degree of variability in an estimate, and thus reflects the consistency (repeatability) of the results generated by a given assay. Robustness refers to the variation across the batches (i.e. runs or plates) performed for a given assay, whereas accuracy is the assays ability to produce results that are identical to the expected (true) values. For each assay, robustness was quantified in terms of the average coefficient of variation (CV) of observed Log DNA amounts across batches. Precision was quantified in terms of the standard deviation of triplicate Cq values, as well as in terms of the R^2^ value associated with the linear regression between the observed and expected Log DNA amount of the serial dilution series. In addition, the slope of the linear regression line allows for assessment of an assays’ accuracy in that a highly accurate assays have a slope equal to one. For example, in Figure 1, the depicted reference gene batches from assay III are highly accurate (slope = 1), but differ in their precision with the observed amount of DNA being less variable and hence more precise in batch A (R^2^ = 0.9963) relative to in batch B (R^2^ = 0.9536).

The resolution of an assay reflects its ability to discriminate between consecutive steps of the dilution series and consequently depends on the precision and accuracy of an assay. We assessed the resolution of each assay by using a pairwise T-test to evaluate whether the average observed Log DNA amounts estimated for each consecutive step in the dilution series were significantly different from each other, as would be expected for assays with sufficient resolution.

### Humpback whale telomere length estimation

#### Outlier detection

Outliers are data points that can be disregarded as irregular observations because they do not follow the distribution of the rest of the data [49]. In the 60 “unknown” humpback whale samples we defined Cq values as outliers if they were outside the linear dynamic range of the standard curve or if the estimated Grubbs’ [50] G-statistic for Cq values or relative telomere length was lower than 1% (Equation 8 in the Appendix).

#### Telomere length estimates across quantitative methods and assays

For each assay, the telomere length of the “unknowns” was estimated using four quantitative approaches, differing with respect to their estimation of amplification efficiency, their inter-batch normalization procedure, and how the raw fluorescence data is baseline corrected.

That is, in addition to the Pfaffl and Ruijter methods described above, telomere length was also estimated by the Pfaffl method where baseline correction was performed in the ABI PRISM and Rotor-Gene software rather than LinRegPCR, as well as by the comparative Cq method [51]. In the comparative Cq method, efficiency is assumed to be constant and similar for both the telomere and reference gene reaction, and inter-batch variation is adjusted by normalization to the Cq value of a calibrator sample included in each batch (Equations 14).

In addition to individual telomere lengths, we estimated the difference between the minimum and maximum telomere length (dMinMax), the average standard deviation of all telomere length estimates (SD), and the ratio between these two measures, which we take to reflect the degree of resolution of the assay.

Finally, the telomere length estimates obtained with different quantitative methods were compared under the rationale that the choice of quantitative method should have minimal effect on telomere length estimates if the assay performs well, that is, large variation in telomere length estimates from a given assay may indicate that the performance of this assay is suboptimal.

## Results

### Primer optimization

Six of the initial reference gene primer pairs were discarded because the failed to amplify, targeted multiple amplicons, or did not amplify consistently across the range of the dilution series ( Additional file 1: Table S1). The 36B4 reference gene primer pair passed the above tests, but was discarded because Sanger sequencing did not provide a satisfactory target sequence. The remaining four primer pairs (two telomere and two reference genes) amplified with adequate specificity and consistency. The linear dynamic range of these primer pairs was approximately similar at 4.5-72.0 ng/reaction for assays I and IV and 2.2-60.0 ng/reaction for assays II and III, with amplification efficiencies decreasing at higher DNA amounts (Table 1, Additional file 2: Figure S1).

### Serial dilution series

#### Non template controls (NTCs)

NTCs are used to detect PCR contamination and to distinguish unintended amplification products such as primer dimers in SYBR green reactions. In the telomere reactions of assay I and IV, the NTC crossed the amplification threshold 10 cycles or more after the most diluted standard DNA (Table 1, Additional file 3: Figure S2A). In assay II and III, the difference in Cq values between the most diluted standard and the NTC was less than 5 cycles. In the reference gene reactions of assay I, the NTCs had Cq values of 40 or more, whereas the NTC Cq values overlapped with the Cq value of the most diluted standard in assay II- IV ( Additional file 3: Figure S2B). However, with respect to the multiplex assays (i.e. II and IV) examination of the melting curve suggests that amplification of the NTCs is not a result of contamination, but caused by primer dimer formation between the telomere and the reference gene primers ( Additional file 4: Figure S3). In Additional file 4: Figure S3C the NTC forms a peak at approximately 87°C that is not observed in the standard and when the qPCRs are performed in singleplex reactions ( Additional file 4: Figure S3A-B). Thus amplification of NTC in assays II and IV should not bias telomere length estimates. Assay III was performed in singleplex and thus for this assay amplification of the NTC may be a result of contamination or primer dimers which could bias telomere length estimates.

#### Amplification efficiency

**Figure 2:**
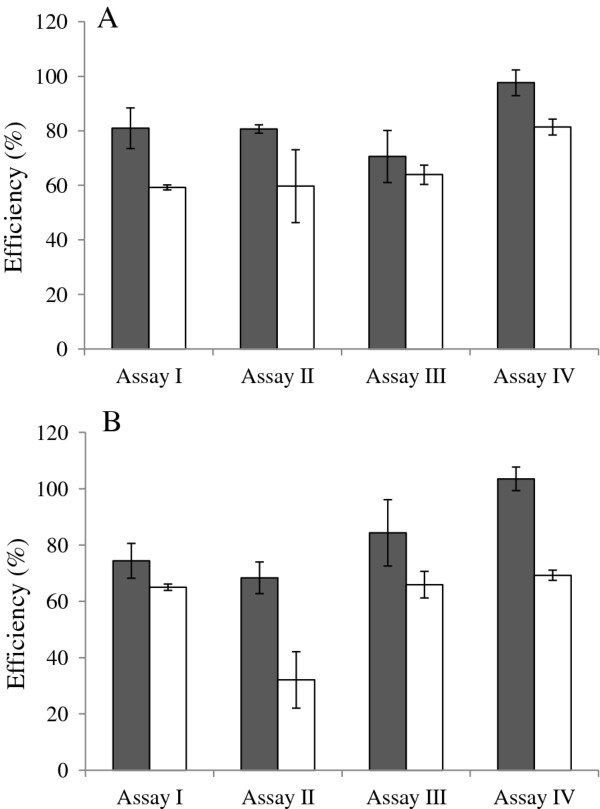
**Average amplification efficiencies and their standard deviation estimated by the standard curve method (dark grey) and the LinRegPCR program (white). A: **the telomere reaction efficiencies. **B: **the reference gene efficiencies.

In both the telomere and the reference gene reactions, estimated amplification efficiencies were higher when determined from the slope of the standard curve compared to the estimates obtained with LinRegPCR (Figure 2). However, efficiency estimates obtained with LinRegPCR were generally less variable among batches within an assay and among the four different assays. Exceptions include assay IV, where the efficiency of the telomere reaction was higher than in the other three assays, and assay II where the efficiency of the reference reaction was very low and variable among batches.

In assay I, there was a strong and highly significant correlation between efficiency and DNA amount in both the telomere and reference gene reactions ( Additional file 5: Table S2). The correlation was strongest for the telomere reactions in which DNA amount per reaction explained more than 75% of the variation in amplification efficiencies with the most diluted standards having higher reaction efficiencies (E = 62.7%) than the undiluted standard (E = 54.8%). None of the other assays exhibited a significant correlation between log exp DNA and amplification efficiency.

#### Precision, robustness, accuracy, and resolution

**Figure 3:**
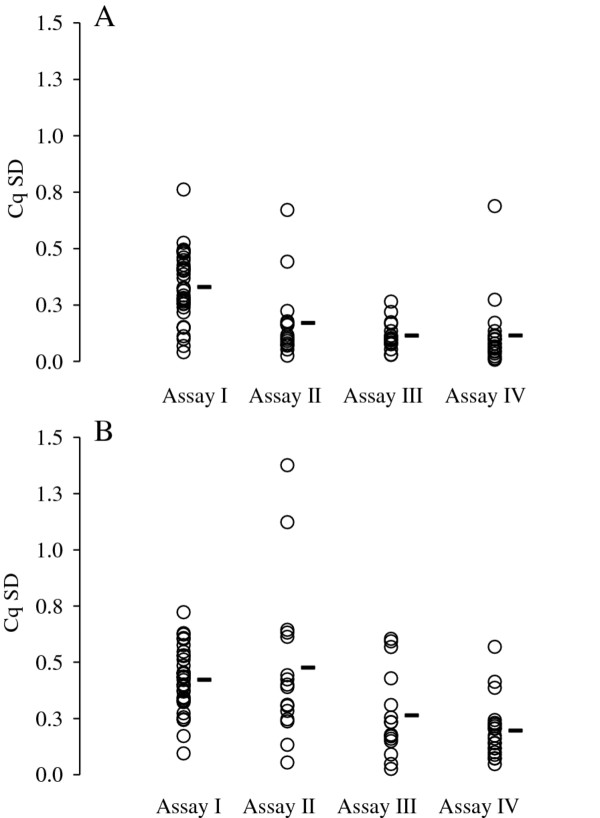
**Precision of each assay quantified in terms of the standard deviation (SD) of Cq values estimated for each triplicate reaction. A: **SD associated with telomere Cq values. **B: **SD associated with reference gene Cq values. Circles denote the SD values estimated for each dilution series and batch and black bars mark the average.

**Figure 4:**
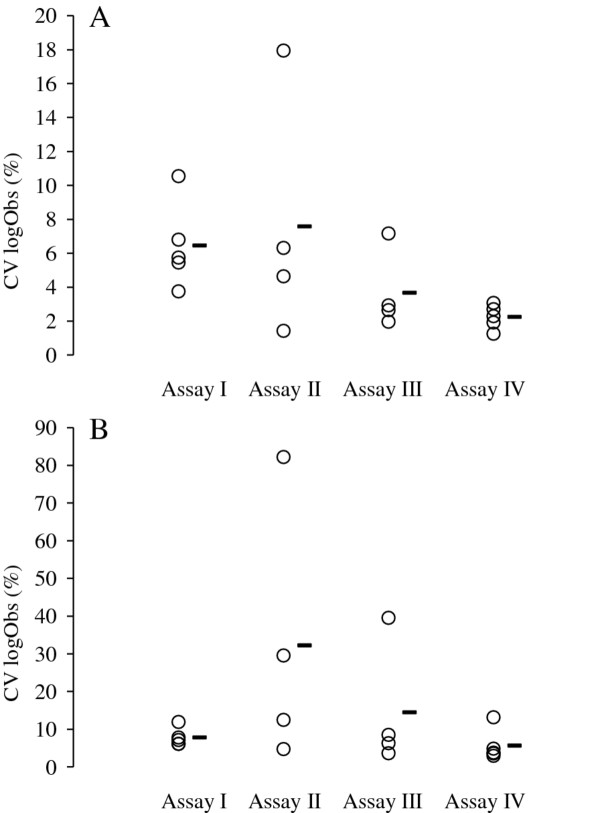
**Robustness of each assay quantified in terms of the coefficient of variation (CV) of logObs estimates across batches. A: **CV associated with telomere logObs values. **B: **CV associated with reference gene logObs values. Circles denote the CV values estimated for each dilution step and black bars mark the average CV for a particular assay.

The precision, robustness and accuracy estimates obtained for each assay is summarized in Figure 3, Figure 4 and Table 2. In general, the telomere reactions were more precise, robust and accurate than the reference gene reactions. More specifically, while the accuracy of all assays is high in the telomere reactions, precision is slightly lower in assay I relative to the other assays. In the reference gene reactions, accuracy varies between assays and quantitative methods, but only assay I has a slope that is significantly different from 1 (two-tailed T-test; *T* = 2.230, *P* = 0.028), suggesting that assay I is inaccurate under these specific conditions. Also, it appears that precision in assay II is slightly lower than in the other assays. Robustness is best for assay IV compared to the other assays.

**Table 2:** **Precision and accuracy of the telomere and reference gene reactions quantified in terms of the standard deviation of Cq values (Cq SD), as well as the correlation coefficient (R**^**2**^**) and the slope of the regression line between expected and observed amount log DNA as illustrated in Figure**1

			**Pfaffl**			**Ruijter**		
**Target**	**Assay**	**Cq SD**	**R**^**2**^	**Slope**	**SD**	**R**^**2**^	**Slope**	**SD**
Telomere	I	0.330	95.6	1.00	0.021	95.8	1.00	0.021
	II	0.171	98.5	0.99	0.019	99.0	0.99	0.016
	III	0.115	99.4	1.00	0.012	99.4	1.00	0.012
	IV	0.116	99.2	1.00	0.012	99.5	1.01	0.010
Reference	I	0.423	95.7	**1.06**	0.027	95.6	1.00	0.021
	II	0.476	92.0	0.98	0.045	93.4	1.02	0.044
	III	0.264	97.0	0.99	0.027	93.4	0.97	0.039
	IV	0.196	96.4	0.97	0.033	97.6	1.02	0.022

Assay I had the lowest resolution with the observed Log DNA amount not being significantly different between the first three consecutive steps of the telomere dilution series, and between the second and third step in the reference gene dilution series (Table 3). Assay II had low resolution between the third and forth step in the reference gene dilution series. Assays III and IV had adequate resolution between all steps of the dilution series.

**Table 3:** Resolution between consecutive steps of the dilution series determined by the P-values of a two-tailed T-test for comparing averages

**Assay**	**Target**	**Batches**	**Exp amount DNA (ng/reaction)**			
			72.0-36.0	36.0-18.0	18.0-9.0	9.0-4.5
I	Tel	7	**0.103**	**0.051**	0.013	0.005
	Ref	7	0.029	**0.052**	0.024	0.022
IV	Tel	4	0.014	0.001	0.000	0.002
	Ref	4	0.010	0.009	0.007	0.012
**Assay**	**Target**	**Batches**	**Exp amount DNA (ng/reaction)**			
			60.0-20.0	20.0-6.7	6.7-2.2	
II	Tel	4	0.013	0.007	0.002	
	Ref	4	0.019	0.018	**0.254**	
III	Tel	4	0.000	0.001	0.000	
	Ref	4	0.032	0.002	0.020	

Bold values are non-significant at the 5% level and indicate that there is no difference between the amounts of DNA observed at two consecutive dilution steps.

**Figure 5:**
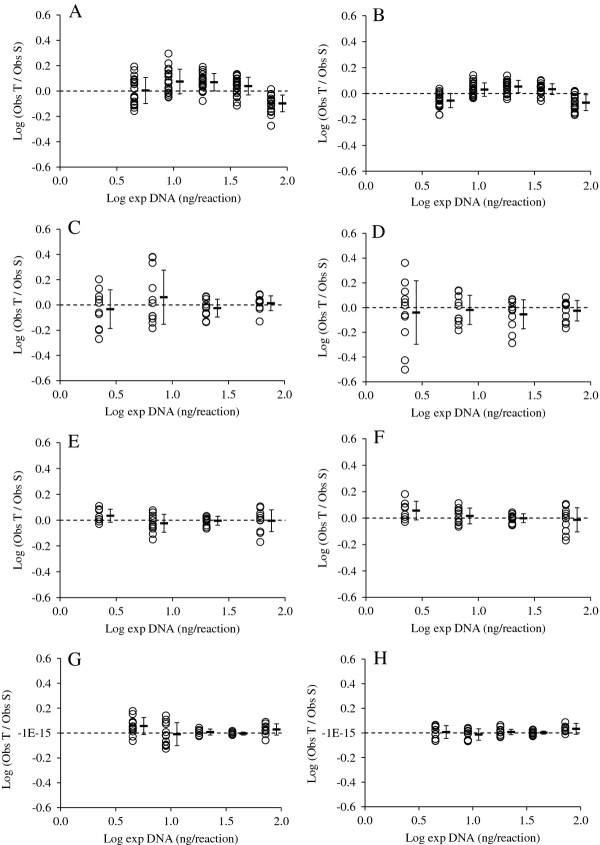
Precision and accuracy of the ratio between telomere and reference in the standards estimated with the Pfaffl method (A, C, E, G) or Ruijter method (B, D, F, H) AB: assay I; CD: assay II, EF: assay III; GH: assay IV.

Finally, the influence of the precision and accuracy of different assays and quantitative methods on telomere length estimates was assessed by plotting the expected amount DNA from the dilution series against the ratio of telomere to reference gene (Figure 5). In assay I, the telomere-reference gene ratio was not constant for different dilution steps with the ratio tending to be lower than expected for the undiluted standards, but higher than expected for the intermediately diluted standards (Figure 5A, B). The telomere-reference gene ratio is more or less constant in assay II, but estimates are associated with a high degree of variability (Figure 5C, D). In assay III, the telomere-reference gene ratio appeared to be constant across dilution steps, except for the most diluted sample in which the ratio was slightly overestimated (Figure 5E, F). In assay IV, the Pfaffl method was less precise than the Ruijter method and appeared to slightly overestimate the telomere-reference gene ratio in the standards with the lowest and highest amount of DNA (Figure 5G, H). Except for this, the ratio was constant across dilution steps and precision was high, particularly at Log exp DNA of 1.56 and 1.26, corresponding to 36 and 18 ng DNA per reaction, respectively.

### Humpback whale telomere length estimation

#### Precision and resolution of telomere length estimates

The number of outliers was low in assay I and IV, but relatively high in assay II and III (Table 4). These latter assays were also associated with the lowest precision of individual telomere length estimates (i.e. a high CV) and the lowest resolution across the range of telomere lengths (i.e. a high SD/(dMinMax)). In assay IV, CV ranged from 0.6% to 30.8% and averaged at approximately 9% depending on quantitative method. Also the average standard deviation of telomere lengths was within 4.9-6.2% of the total range of telomere lengths, which suggest a 2–4 times higher (better) resolution than the other assays, depending on the quantitative method used.

**Table 4:** Basic characteristics of telomere lengths in humpback whales determined by different assays and quantitative methods

			**T/S**				**SD**			**CV%**			
**Assay**	**Method**	**N**	**Av**	**Min**	**Max**	**dMinMax**	**Av**	**Min**	**Max**	**Av**	**Min**	**Max**	**SD/(dMinMax)%**
I	Pfaffl LR	58	1.05	0.38	1.89	1.52	0.16	0.01	0.71	19.0	1.8	52.0	10.9
	Pfaffl RG	60	1.29	0.38	2.42	2.04	0.22	0.01	0.47	15.8	1.6	47.8	11.0
	Ruijter	60	1.36	0.36	2.60	2.24	0.25	0.02	0.81	17.1	4.7	41.2	11.2
	ddCq	58	1.19	0.29	2.17	1.88	0.23	0.01	0.80	17.9	1.6	49.5	12.4
II	Pfaffl LR	54	0.84	0.15	1.73	1.57	0.19	0.01	0.71	21.4	3.3	72.1	12.1
	Pfaffl RG	49	0.77	0.15	1.80	1.65	0.31	0.03	1.86	35.5	6.0	131.0	19.1
	Ruijter	51	0.73	0.15	1.51	1.36	0.16	0.01	0.58	21.8	3.3	72.1	11.9
	ddCq	54	3.38	0.07	20.46	20.39	1.21	0.02	9.76	30.3	3.6	93.2	5.9
III	Pfaffl LR	54	0.80	0.24	1.39	1.15	0.20	0.00	1.22	25.1	0.2	89.1	17.1
	Pfaffl RG	55	0.87	0.19	1.62	1.43	0.31	0.04	1.12	49.6	4.5	372.4	21.6
	Ruijter	56	0.82	0.17	1.40	1.23	0.21	0.01	1.25	26.4	0.6	89.1	17.3
	ddCq	54	0.73	0.19	1.94	1.75	0.22	0.00	1.39	30.6	0.1	111.9	12.3
IV	Pfaffl LR	59	1.58	0.44	2.87	2.43	0.14	0.01	0.42	8.7	1.3	29.6	5.7
	Pfaffl RG	59	1.61	0.45	3.15	2.70	0.14	0.02	0.51	9.0	0.6	30.8	5.3
	Ruijter	59	1.54	0.45	2.62	2.16	0.13	0.01	0.44	8.8	1.3	29.6	6.2
	ddCq	60	1.53	0.45	2.93	2.48	0.12	0.01	0.31	8.2	1.3	27.2	4.9

N = number of samples after outlier removal; T/S = Telomere length; Av = average; Min = minimum T/S; Max = maximum T/S; dMinMax = difference between minimum and maximum T/S; SD = standard deviation of T/S estimates; CV% = the coefficient of variation of T/S estimates in percent; SD/(dMinMax) = the ratio in percent between SD and the difference between minimum and maximum T/S. Pfaffl LR = the Pfaffl method with baseline corrected in LinRegPCR; Ruijter = the Ruijter method; ddCq = the comparative Cq method; Pfaffl RG = the Pfaffl method with baseline corrected in Rotor-Gene or ABI software. See text for details.

#### Effect of quantitative method

For each assay, the telomere length of the “unknowns” was estimated using four quantitative approaches differing with respect to their estimation of amplification efficiency, their inter-batch normalization procedure, and how the raw fluorescence data was baseline corrected.

In assay I and IV, the correlation between telomere length estimates obtained with different quantitative methods was generally good with R^2^ = 81.9-95.6% and R^2^ = 87.7-96.6% for assays I and IV, respectively ( Additional file 6: Table S3). In contrast, telomere length estimates based on the data generated in assay II and III exhibited greater variation across quantification methods, suggesting that the results generated from these assays should be interpreted with caution. This was most prominent for the assay II data in which telomere lengths correlated with as little as R^2^ = 11.0% when estimated by the methods of Livak and Schmittigen (2001) and Ruijter (2009), respectively. If we assume that assay IV provide the most reliable results it appears that although the estimated amplification efficiencies differed by as much as 20% between the Pfaffl and Ruijter methods (Figure 2) the correlation between telomere lengths estimates was still high (R^2^ = 96.6%), and similar or marginally better than telomere length estimates obtained using different baseline correction methods (i.e. R^2^ = 91.9%).

## Discussion

The qPCR method for telomere length estimation has been criticized for being difficult to optimize, extremely sensitive to technical errors, and for being misused in assessments of telomere dynamics in model and non-model species [30-32]. This criticism coincides with an increasing awareness of the requirement and wide adoption of a general set of guidelines for experimental, analytical and reporting steps of qPCR in general [26-28]. Our study indicate that this criticism is not unjust, but in doing so also identifies some of the factors that may cause variation in qPCR experiments and exemplifies a series of quality controls that may aid in detecting suboptimal data (Table 5). Importantly, we show that it is possible to obtain high precision and accuracy in relative telomere length estimates by qPCR, but it requires extensive optimization and quality control at all experimental and analytical steps.

**Table 5:** Summary of quality control steps and overall assay performance for the telomere (T), reference (S), and telomere length estimates (T/S)

**Criteria**	**Target**	**Assay I**	**Assay II**	**Assay III**	**Assay IV**	**Figure/Table**
**Serial dilution series**						
*LDR*	T	Borderline	Within range	Within range	Within range	Figure 2
	S	Borderline	Within range	Within range	Within range	
*NTC*	T	OK	Borderline	Borderline	OK	Figures 3, 4
	S	OK	Overlap*	Overlap	Overlap*	
*Magnitude E*	T	Intermediate	Intermediate	Intermediate	High	Figure 1
	S	Intermediate	Low	Intermediate	Intermediate	
*Interbatch E*	T	Constant	Variable	Constant	Constant	Figure 1
	S	Constant	Variable	Constant	Constant	
*Dilution E*	T	Variable E	No change	No change	No change	Table 3
	S	Variable E	No change	No change	No change	
*Precision*	T	Intermediate	High	High	High	Figure 5, Table 4
	S	Intermediate	Low	Moderate	High	
*Accuracy*	T	High	High	High	High	Table 4
	S	High	Intermediate	High	Intermediate	
*Resolution*	T	Low	High	High	High	Table 5
	S	Intermediate	Intermediate	High	High	
*Precision*	T/S	Intermediate	Low	Intermediate	Intermediate	Additional file 3: Figure S2
*Accuracy*	T/S	Low	Low	High	High	Additional file 3: Figure S2
**"Unknown" samples**						
*Outliers*	T/S	Few	Many	Many	Few	Additional file 1: Table S1
*Resolution*	T/S	Intermediate	Low	Low	High	Additional file 1: Table S1
*Quantitative method*	T/S	High	Low	Intermediate	High	Additional file 5: Table S2
*Inter-assay correlation*	T/S	Low	Low	Low	Intermediate	Additional file 4: Figure S3
**Assay evaluation**		Intermediate	Worst	Intermediate	Best	

### Assay evaluation

#### Assay I

The performance of assay I and the other three assays is summarized in Table 5. To judge from the efficiency of telomere and reference gene reactions and the R^2^ of the standard curves, assay I generally performed well. However, closer examination suggests that, under the given circumstances, this assay is associated with moderate levels of precision and a resulting low resolution between dilution steps. In addition, the efficiency estimated with the LinRegPCR software tended to vary across dilution steps, with the most diluted standards having significantly higher efficiency than the undiluted standards. The correlation between DNA amount and efficiency was stronger for telomere than the reference gene, indicating that telomere length may be underestimated for individuals with long telomeres (i.e. high amount of telomere DNA). This suspicion was strengthened by the observation that the ratio between telomere and reference gene was lower at high amounts of DNA relative to intermediate amounts.

#### Assay II

Assay II was the worst performing of the four assays with low to moderate and highly variable amplification efficiencies. This was reflected in the low precision and accuracy of the reference gene reactions and the telomere-reference gene ratio, the high fraction of outliers, the relatively low resolution among individual telomere length estimates, as well as the correlation in telomere lengths between different quantitative methods and with the other assays. Interestingly, the telomere reactions were found to have high precision, accuracy and resolution despite the fact that the NTCs indicated the potential presence of contamination or primer dimers.

#### Assay III

In assay III amplification efficiencies were consistent across batches and precision, accuracy, and resolution moderate to high in both the telomere and reference gene reactions. However, the Cq of the telomere and reference gene NTCs either overlapped or were proximate to the Cq of the most diluted standards, indicating the presence of contamination or primer-dimers. Moreover, the assay was characterized by a relatively high number of outliers, high CVs and relatively low resolution among individual telomere length estimates, and only intermediate correlation between the telomere length estimates obtained by different quantitative methods. This serves to illustrate that high amplification efficiencies and a good fit of the standard curve is not a guarantee that an assay will produce reliable results.

#### Assay IV

This assay was the best performing of the four assays, having moderate to high precision, accuracy and resolution both when examining the telomere and reference genes in isolation as well as for estimated telomere lengths. This was also reflected in a high correlation between telomere lengths estimates obtained with different quantitative methods. Assay IV thus fulfill our criteria for a good assay.

### Factors affecting telomere length estimates

Our evaluation of assay performance serves to identify some of the factors that may affect the precision and accuracy of qPCR in general, and telomere length estimation in specific.

#### Telomere and reference gene primers

A significant obstacle to qPCR amplification of telomeres was the difficulty associated with designing primers that hybridize to the telomere repeats without forming primer dimer derived products. Seemingly, this problem was overcome by Cawthon who designed a set of primers for telomere amplification in humans in which build-in mismatches ensures higher primer specificity to the telomere repeat relative to the opposite primer [24,25,52]. As the telomere repeat is similar for all vertebrates [2] these primers should be generic. We found that the telomere primers used in assay II-IV amplified with high precision and accuracy, whereas precision was lower for the primers used in assay I. Moreover, in this assay amplification efficiencies for the telomere reactions appeared to be affected by the amount of DNA in the reaction. The telomere primers used in assay I differ from those of assay II, III and IV in that the latter amplify a product of fixed length with a well-defined melting profile, allowing for subsequent evaluation of each reaction. Hence, the telomere primers described in Cawthon [24] appear most suitable for telomere length estimation – also in situations where limited access to multiplex qPCR platforms and software necessitate the use of the singleplex method.

The interspecific use of qPCR primers may be more problematic for the reference gene where potential interspecific differences in occurrence of e.g. SNPs, splice variants, and pseudo genes can substantially affect primer specificity and hence amplification efficiency. Primers with high efficiency have higher precision (less variability) than primers with lower efficiency [53]. The difficulties associated with obtaining good reference gene primers is illustrated by the fact that only two of the initial set of 8 primers passed quality control and that the performance of one of these — the assay I reference gene primers — later turned out to be significantly affected by DNA amount. Hence in addition to initial optimization and quality control it is essential to perform subsequent evaluation once data has been generated.

Moreover, because the single copy reference genes typically amplify at a higher cycle number than the target genes, and because high Cq values usually are associated with larger variability [47], reference gene reactions may per default introduce a certain degree of variability into telomere length estimates. In the present study, the Cq’s of the reference gene reactions were typically 15–20 cycles higher than the telomere reactions, and the precision of the reference gene was always lower than that of the telomere. Cycle number may be lowered, and hence precision increased, by increasing the amount of template DNA per reaction. However, the optimal amount likely differs for different assays, even when the same primers are used. For example, while precision in assay III appear to be highest at 20 ng template DNA per reaction, the precision of assay IV may, in retrospective, have been further increased by increasing the amount of template DNA from 20 ng to 36 ng per reaction. Moreover, since a further increase to 72 ng template DNA per reaction causes a reduction in precision, the optimal range of DNA template amount for telomere length measurement may be rather narrow. Consequently, we suggest that optimal amount of template DNA is identified by first using a serial dilution series with a high fold difference between dilution steps and subsequently exploring the optimal range in more detail by a more narrow serial dilution series spanning a lower fold difference in amount template DNA.

To ensure high precision and reliability of results, Bustin and co-authors [26] recommended the use of at least three reference genes and that the performance of these are tested using e.g. geNorm [54]. The present telomere length estimations are based on normalization to a single reference gene, and although the reference gene primer pair used in assay II-IV has been validated and widely used in molecular studies of cetaceans [38], future applications of the qPCR approach to telomere length measurement may benefit from the inclusion of more reference genes.

#### Experimental design

Experimental design includes the choice of qPCR platform and consumables, whether to perform the telomere and reference gene reactions in singleplex or multiplex, which reagents to use, and the choice of standards for standard curves and inter-ba tch calibration.

Platforms and their specific consumables may differ in their abilities to adjust the temperature of the qPCR reaction and their accuracy in measuring the fluorescence signal from each reaction. Here, assay I was conducted using an ABI PRISMTM 7000 Sequence Detection System while assays II-IV were performed in a QIAGEN Rotor-Gene Q (former Corbett Rotor-Gene 6000) qPCR cycler. Preliminary results suggest that qPCRs performed in the ABI platform are subject to edge-effects in which temperature differences across the heating block cause variation in reaction amplification efficiencies (Olsen MT, in prep). Although we attempted to reduce this effect by not using the wells along the edge of the plate minor variations in temperature across the heating block may have contributed to reduce the precision of assay I.

Multiplexing may increase the precision of telomere length estimates by removing variability between the telomere and reference gene reactions caused by e.g. pipetting [24]. However if not sufficiently optimized, multiplexing can lead to the formation of primer dimers and/or cause inhibition of one or both reactions as a consequence of competition for reagents. The low amplification efficiencies of the reference gene reactions in assay II and the resulting variability of telomere length estimates produced with this assay is likely caused by such competition between the telomere and reference gene primers. If so, this inhibition is likely to be caused by suboptimal concentrations of reagents and/or primers in that all other aspects of assay II were similar to assay IV which worked fine.

The use of standard curves to estimate telomere lengths and adjust for inter-batch variation relies on the assumption that standards have similar amplification efficiencies as the unknowns. We sought to reduce potential differences in efficiency by constructing standard curves from a pool of DNA extracted by the same method and from the same tissue and species as the unknowns. Further, the duration of the entire assay was short (5–21 days) and the standards kept in fridge in order to minimize potential DNA degradation caused by freeze-thawing and/or prolonged storage. Similarity in amplification efficiencies was controlled by estimating individual efficiencies with the LinRegPCR software. Preliminary tests suggest that standards retained their precision and accuracy even after more than two months of storage in the fridge.

Finally, it is important to realize that all qPCR experiments are associated with a certain degree of internal stochasticity and that some inter-batch and inter-assay variation is unavoidable because different batches and assay are carried out at different times and/or by different persons [28].

#### Data processing and analysis

In qPCR, there are two aspects of amplification efficiency; one is the potential influence of primer efficiency on accuracy and precision as discussed above, and the other the influence of inferred amplification efficiency on data analysis. There is currently little consensus about the best method to determine the true amplification efficiency of a reaction, and new methods are continuously reported which claim superiority to existing methods [45,46,51,54-58]. For assay IV, our comparison of telomere length estimates obtained with different methods show that even though efficiency estimates obtained from the standard curve (the Pfaffl method) and LinRegPCR (the Ruijter method) differed by 10-40%, the resulting telomere length estimates varied by less than 5%. Thus, if a given primer set/assay has documented high precision and meets the other quality criteria, the inferred efficiency of this primer pair may be of less concern in telomere length measurements, as also suggested by Regier and Frey [59]. Indeed, the comparisons between the ddCq, Ruijter, and Pfaffl methods show that disregarding efficiency all together (the ddCq method) only increased inter-method variation in telomere length estimates to 10%, approximately the same level of variation introduced by using different baseline correction methods (Pfaffl LR vs Pfaffl RG).

#### Inappropriate technology

Finally, the choice of technology for telomere length measurement may significantly affect estimates. The two most common methods for telomere length estimation are the telomere restriction fragment (TRF) and qPCR methods. We chose the latter because is it supposedly quicker, more sensitive, and less technical than the TRF method and thus allows for a higher throughput. The higher throughput also serves to reduce the costs – particularly if the qPCR multiplex method is used. Moreover, the qPCR method is associated with less stringent requirements on DNA amount and quality [60], which is crucial when determining telomere length in free-ranging species such as humpback whales where sampling is remotely and skin tissue the only source of DNA. Recently, Kimura and Aviv [61] presented a new method for telomere length estimation based on dot blot analysis and the SYBR Dx DNA Blot Stain. This method does not require normalization to a reference gene and is performed in “multiplex”, reducing the potential variation caused by pipetting errors. Hence, the dot blot method may prove to be a less error-prone complement or alternative to the qPCR method, although its applicability for telomere length estimation in non-model species is still to be assessed.

## Conclusion

Telomeres hold great promise as markers of individual life history, health, and fitness. The above should serve to illustrate some of the potentials and pitfalls associated with telomere length measurement by the qPCR method. As noted by Kimura and Aviv [61], the measurement of true telomere length is a standard that can only be approached asymptotically and it will never be possible to fully remove experimental, biological and stochastic errors. However, by recognizing its limitations and aiming at reducing error by careful experimental design and rigorous quality control at all stages of analysis, the qPCR method may nevertheless be good to obtain relative trends, especially for non-model species where experimental alternatives are lacking. It is our hope that the analyses and quality control summarized in Table 5 may serve as a preliminary set of guidelines for a more rigorous quality control of telomere length estimates by qPCR, as well as inspire to the continuous use of telomeres as a proxy for biological age and life histories in model and non-model species.

## Appendix

Quantification with the Pfaffl method [48]

1. A standard curve is produced by plotting Log DNA amount against Cq to give a linear regression line. The line is described by y=ax+b, where y = Cq; x = Log expected DNA amount; a = the slope; b = the y-intercept

2. Amplification efficiency of the standards are estimated from the standard curve as $E=10^{\wedge }\left(-1/\text{slope}\right)$ and assumed to be similar to the efficiency of the “unknown” samples.

3. The observed amount of telomere is estimated as $T=E^{\wedge }\left(\text{Cq calibrator}–\text{Cq sample}\right)$ which corresponds to $T=10^{\wedge }\left(\left(\text{Cq calibrator}–\text{Cq sample}\right)/\text{slope}\right)$ or expressed in terms of the linear regression components $T=10^{\wedge }\left(\left(b-y\right)/a\right)$. The observed amount of reference gene (S) is estimated in the same way.

4. Relative telomere length (T/S) is estimated as the ratio between amount telomere (T) and reference gene (S).

Quantification with the Ruijter method [46]

5. The starting concentration (N0) is determined by $N0=\text{Nt}/\left(E^{\wedge }\text{Cq}\right)$, where Nt = is the threshold line and E = the average amplification efficiency of all reactions.

6. We converted estimates of N0 into observed amount of telomere (T) using $T=10^{\wedge }\left(\left(\text{log N}0–\text{intercept}\right)/\text{slope}\right)$ which corresponds to $T=10^{\wedge }\left(\left(y-b\right)/\text{a}\right)$ where a and b is the slope and intercept of the linear regression between expected Log DNA amount and Log N0. The observed amount of reference gene (S) is estimated in the same way.

7. Relative telomere length (T/S) is estimated as the ratio between amount telomere (T) and reference gene (S).

Grubbs’ outlier detection [50]

8. Grubbs’ outlier detection test in which $G=\left|\left(\text{Cq}–\text{mean Cq}\right)/\text{SD}\right|$, where G is the test-statistic and SD the standard deviation. G > 5% are included in the analysis; G = 1-5% are defined as stragglers but kept in the analysis; G < 1% are outliers and discarded.

## Competing interests

The authors declare no financial or non-financial competing interests.

## Authors’ contributions

MTO participated in conceiving and designing the study, carried out qPCR experiments and statistical analyses, and wrote the manuscript. MB participated in conceiving and designing the study, extracted DNA, and performed sequencing analyses. JR supplied all humpback whale samples and biological data. PJP conceived of the study, participated in its design, and helped draft the manuscript. All authors read and approved the final manuscript.

## Supplementary Material

Additional file 1 **Table S1. **Reference gene primers tested. BA = Bérubé and Aguilar [38]; CW 2006 = Callicot and Womack [52]; RC 2009 = Cawthon [24]. Note that all reference gene primers used in assays II-IV had a CG-clamp (CGGCGGCGGGCGGCGCGGGCTGGGCGG) attached to increase annealing temperature as described in Cawthon (2009). * Caution; this primer pair was used in assay I in which primer efficiency was found to correlate with DNA concentration. Click here for file

Additional file 2 **Figure S1. **Example standard curve for the telomere portion of assay II. In A. it is clear that amplification efficiency decrease in reactions with log DNA above 1.78 (20 ng DNA/reaction) causing deviation from linearity of the standard curve. As illustrated in B. the the linear dynamic range of the telomere primer is within log 1.778-0.347 DNA, corresponding to 60–2.2 ng DNA per reaction. Note how the difference in the slopes of the standard curves in A and B affects the estimated amplification efficiency. In A. the amplification efficiency is E = 2.050 (105.0%) whereas it is E = 1.787 (78.7%) in B. Relationship between and observed “variable” (Ct, N0, fluorescence) against known concentration in a dilution series. Click here for file

Additional file 3 **Figure S2. **Amplification of non template controls (NTC) in the four assays relative to the most diluted standard in the serial dilution series in telomere (A) and reference gene (B). Circles denote the Cq value of individual NTC reactions and bars mark the average Cq and standard deviation of the most diluted standards. Note that the overlap between NTC and standards in the reference gene reactions of multiplex assay II and IV result from telomere-reference gene primer dimers as shown in Supplementary **Figure 3A-C.** Click here for file

Additional file 4 **Figure S3. **Representative melting curves for the standard and NTC reactions. Black line is the most diluted standard and stippled line the NTC. A: the telomere reaction in assay III where the NTC starts amplifying a few cycles after the most diluted standard. B: the reference gene reaction in assay III where the NTC and standard overlap. C: the multiplex reaction in assay II in which telomere-reference gene primer dimers are producing a peak at approximately 87°C in the NTC but not in the standard. Click here for file

Additional file 5 **Table S2. **Linear regression and correlation between expected log amount DNA in standards and their amplification efficiencies as estimated in LinRegPCR. Bold values are significant at the 5% level. Slope = slope of the regression line; Intercept = intercept of regression line reflecting the hypothetical maximum efficiency in percent. Click here for file

Additional file 6 **Table S3. **Percent correlation (R^2^) between telomere length estimates obtained by different quantitative methods. Pfaffl LR = the Pfaffl method with baseline corrected in LinRegPCR; Ruijter = the Ruijter method; ddCq = the comparative Cq method; Pfaffl RG = the Pfaffl method with baseline corrected in Rotor-Gene or ABI software. See text for details. Click here for file
