# Supplementary material for: Empirical evaluation of humpback whale telomere length estimates; quality control and factors causing variability in the singleplex and multiplex qPCR methods
Source: BMC Genet. 2012 Sep 6;13:77. doi: 10.1186/1471-2156-13-77 (PMC3489520; doi:10.1186/1471-2156-13-77)
Supplement: Additional file 1 — Table S1. Reference gene primers tested. BA = Bérubé and Aguilar [38]; CW 2006 = Callicot and Womack [52]; RC 2009 = Cawthon [24]. Note that all reference gene primers used in assays II-IV had a CG-clamp (CGGCGGCGGGCGGCGCGGGCTGGGCGG) attached to increase annealing temperature as described in Cawthon (2009). * Caution; this primer pair was used in assay I in which primer efficiency was found to correlate with DNA concentration. [file 1471-2156-13-77-S1.doc]

**Supplementary Table 1** Reference gene primers tested

| Gene | Primer name | Primer sequence (excl. CG-clamp) 5’-3’ | Length (bp) | Source | Comment |
| --- | --- | --- | --- | --- | --- |
| Esterase D | Mn.ACTE8F | GCATTTGCTCCAATCTGCAACCCA | 99 | This study | Several amplicons |
|  | Mn.ACTE8R | TTTGCCCCAAGGACAGAGCACT |  |  |  |
| Actin | Mn.ACTI1R | CATAGTGGCGAACAAGACAG | 169 | This study | No amplicon |
|  | Mn.ACTI1F | TCTCTCAAATAGCAGAGAACCCT |  |  |  |
| Fibrinogen | Mn.FGGI6aF | ACCATCAGTACAAGAGTCAGCA | 99 | This study | No amplicon |
|  | Mn.FGGI6aR | ACTCCAGAACACCAGCTTGA |  |  |  |
| Fribinogen | Mn.FGGI6bF | TCTCTCCTTTTGCCCGTGCAAT | 106 | This study | Not consistent |
|  | Mn.FGGI6bR | CCACACTGCCATCAAGCCTCTAAT |  |  |  |
| Albumin | albuF | AAATGCTGCACAGAATCCTTG | 98 | RC 2009 | Good |
|  | albdR | GAAAAGCATGGTCGCCTGTT |  |  |  |
| Beta-globin | hbguF | CTTCATCCACGTTCACCTTG | 106 | RC 2009 | Several amplicons |
|  | hbgdR | GAGGAGAGGTCTGCCGTT |  |  |  |
| α-lactalbumin | LacII.F | CCAAAATGATGTCCTTTGTC | 540 | BA 1998 | Good* |
|  | Lac1.R | CTCACTGTCACAGGAGATGT |  |  |  |
| Acidic ribosomal | 36B4F | ACTGGTCTAGGACCCGAGAAG | 76 | CW 2006 | No sequence |
| phosphorprotein | 36B4R | TCAATGGTGCCTCTGGAGATT |  |  |  |

BA = Bérubé and Aguilar [38]; CW 2006 = Callicot and Womack [52]; RC 2009 = Cawthon [24]. Note that all reference gene primers used in assays II-IV had a CG-clamp (CGGCGGCGGGCGGCGCGGGCTGGGCGG) attached to increase annealing temperature as described in Cawthon (2009). * Caution; this primer pair was used in assay I in which primer efficiency was found to correlate with DNA concentration.
