# Supplementary figures and images for: Empirical evaluation of humpback whale telomere length estimates; quality control and factors causing variability in the singleplex and multiplex qPCR methods
Source: BMC Genet. 2012 Sep 6;13:77. doi: 10.1186/1471-2156-13-77 (PMC3489520; doi:10.1186/1471-2156-13-77)

Supplementary Figure 1

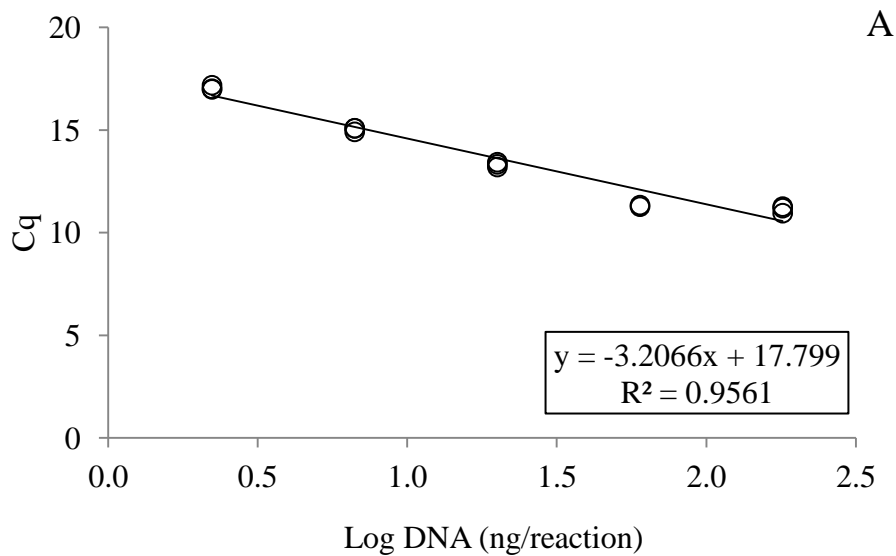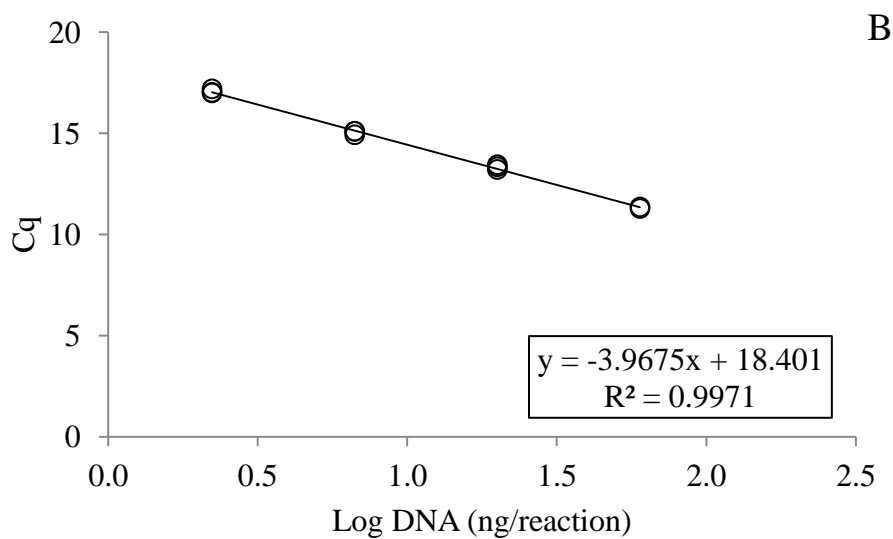

Supplement: Additional file 2 — Figure S1. Example standard curve for the telomere portion of assay II. In A. it is clear that amplification efficiency decrease in reactions with log DNA above 1.78 (20 ng DNA/reaction) causing deviation from linearity of the standard curve. As illustrated in B. the the linear dynamic range of the telomere primer is within log 1.778-0.347 DNA, corresponding to 60–2.2 ng DNA per reaction. Note how the difference in the slopes of the standard curves in A and B affects the estimated amplification efficiency. In A. the amplification efficiency is E = 2.050 (105.0%) whereas it is E = 1.787 (78.7%) in B. Relationship between and observed “variable” (Ct, N0, fluorescence) against known concentration in a dilution series. [file 1471-2156-13-77-S2.pdf]

**Supplementary Figure 2**

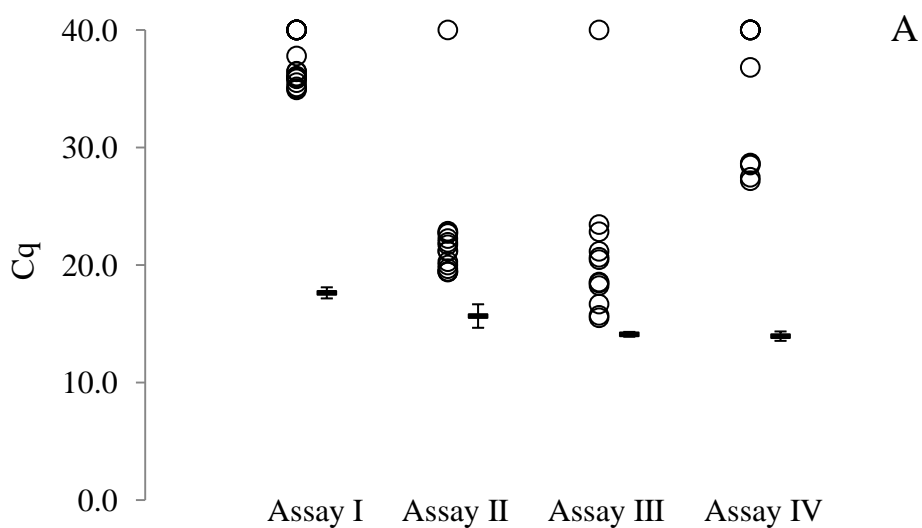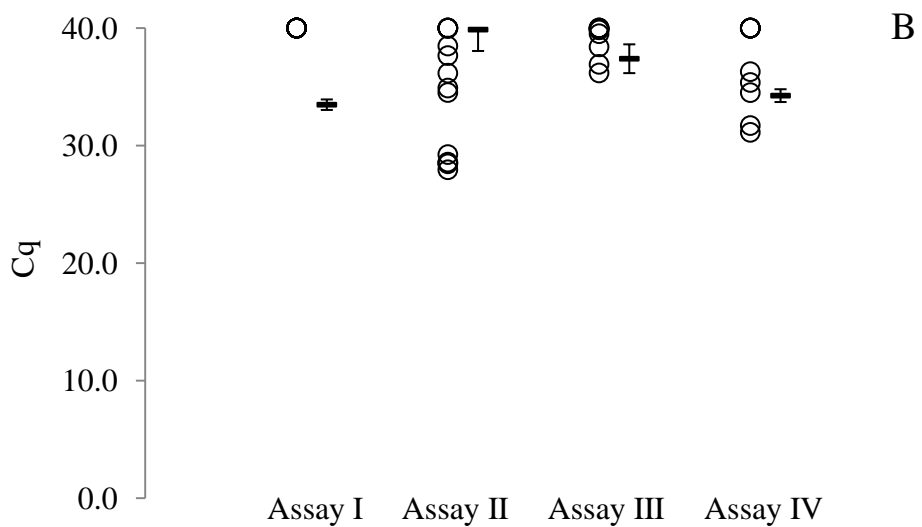

Supplement: Additional file 3 — Figure S2. Amplification of non template controls (NTC) in the four assays relative to the most diluted standard in the serial dilution series in telomere (A) and reference gene (B). Circles denote the Cq value of individual NTC reactions and bars mark the average Cq and standard deviation of the most diluted standards. Note that the overlap between NTC and standards in the reference gene reactions of multiplex assay II and IV result from telomere-reference gene primer dimers as shown in Supplementary Figure 3A-C. [file 1471-2156-13-77-S3.pdf]

**Supplementary Figure 3**

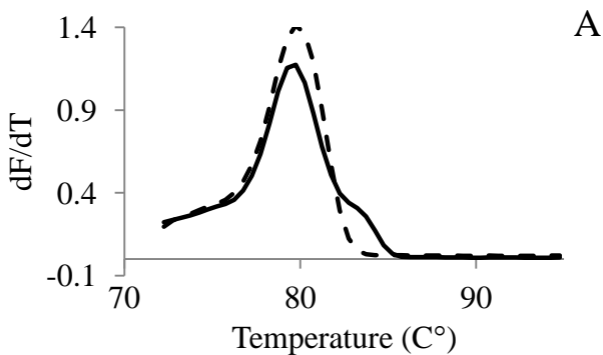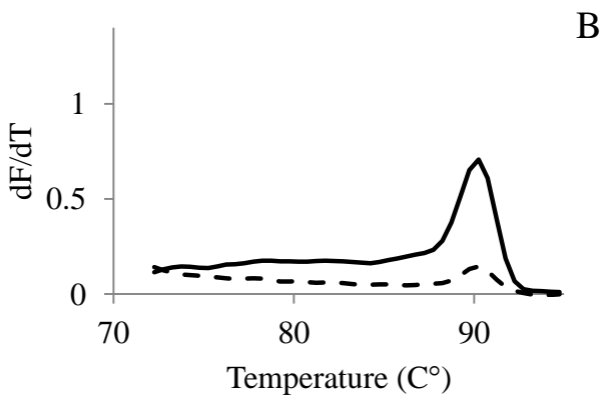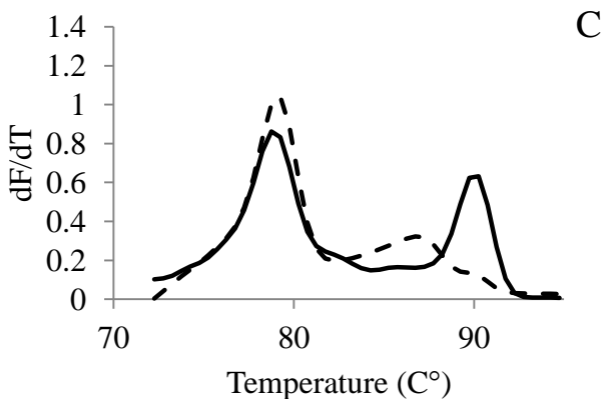

Supplement: Additional file 4 — Figure S3. Representative melting curves for the standard and NTC reactions. Black line is the most diluted standard and stippled line the NTC. A: the telomere reaction in assay III where the NTC starts amplifying a few cycles after the most diluted standard. B: the reference gene reaction in assay III where the NTC and standard overlap. C: the multiplex reaction in assay II in which telomere-reference gene primer dimers are producing a peak at approximately 87°C in the NTC but not in the standard. [file 1471-2156-13-77-S4.pdf]
