# Supplementary material for: Empirical evaluation of humpback whale telomere length estimates; quality control and factors causing variability in the singleplex and multiplex qPCR methods
Source: BMC Genet. 2012 Sep 6;13:77. doi: 10.1186/1471-2156-13-77 (PMC3489520; doi:10.1186/1471-2156-13-77)
Supplement: Additional file 5 — Table S2. Linear regression and correlation between expected log amount DNA in standards and their amplification efficiencies as estimated in LinRegPCR. Bold values are significant at the 5% level. Slope = slope of the regression line; Intercept = intercept of regression line reflecting the hypothetical maximum efficiency in percent. [file 1471-2156-13-77-S5.doc]

**Supplementary Table 2** Linear regression and correlation between expected log amount DNA in standards and their amplification efficiencies as estimated in LinRegPCR. Bold values are significant at the 5% level.

| Assay | Target | Slope | Intercept | *R^2^* | *F* | *P* |
| --- | --- | --- | --- | --- | --- | --- |
| I | Telomere | -6.13 | 66.6 | 0.759 | 103.84 | 0.000 |
|  | Reference | -2.15 | 67.8 | 0.271 | 12.27 | 0.001 |
| II | Telomere | 0.05 | 55.1 | 0.045 | 0.66 | 0.430 |
|  | Reference | 0.05 | 27.1 | 0.081 | 1.24 | 0.285 |
| III | Telomere | 1.95 | 61.8 | 0.057 | 0.85 | 0.371 |
|  | Reference | 0.49 | 65.4 | 0.003 | 0.04 | 0.838 |
| IV | Telomere | -0.03 | 85.0 | 0.116 | 2.37 | 0.141 |
|  | Reference | 0.02 | 64.7 | 0.112 | 2.28 | 0.149 |

Slope = slope of the regression line; Intercept = intercept of regression line reflecting the hypothetical maximum efficiency in percent
