# Supplementary material for: Empirical evaluation of humpback whale telomere length estimates; quality control and factors causing variability in the singleplex and multiplex qPCR methods
Source: BMC Genet. 2012 Sep 6;13:77. doi: 10.1186/1471-2156-13-77 (PMC3489520; doi:10.1186/1471-2156-13-77)
Supplement: Additional file 6 — Table S3. Percent correlation (R2) between telomere length estimates obtained by different quantitative methods. Pfaffl LR = the Pfaffl method with baseline corrected in LinRegPCR; Ruijter = the Ruijter method; ddCq = the comparative Cq method; Pfaffl RG = the Pfaffl method with baseline corrected in Rotor-Gene or ABI software. See text for details. [file 1471-2156-13-77-S6.doc]

**Supplementary Table 3 Percent correlation (R^2^) between telomere length estimates obtained by different quantitative methods**

| Comparison | Assay I | Assay II | Assay III | Assay IV |
| --- | --- | --- | --- | --- |
| Pfaffl LR vs. Ruijter | 81.9 | 76.4 | 81.2 | 96.6 |
| Pfaffl LR vs. ddCq | 95.6 | 31.1 | 66.7 | 91.5 |
| ddCq vs. Ruijter | 85.2 | 11.0 | 37.6 | 87.7 |
| Pfaffl LR vs. Pfaffl RG | 88.0 | 51.4 | 71.5 | 91.8 |
| Average | 87.7 | 42.5 | 64.3 | 91.9 |

Pfaffl LR = the Pfaffl method with baseline corrected in LinRegPCR; Ruijter = the Ruijter method; ddCq = the comparative Cq method; Pfaffl RG = the Pfaffl method with baseline corrected in Rotor-Gene or ABI software. See text for details.
